# Incidence and predictors of asymptomatic atrial fibrillation in patients older than 70 years with complete atrioventricular block and dual chamber pacemaker implantation

**DOI:** 10.3325/cmj.2011.52.61

**Published:** 2011-02

**Authors:** Vjekoslav Radeljić, Nikola Pavlović, Šime Manola, Diana Delić-Brkljačić, Hrvoje Pintarić, Dubravko Petrač

**Affiliations:** Department of Cardiology, Sisters of Mercy University Hospital, Zagreb, Croatia

## Abstract

**Aim:**

To evaluate predictors of asymptomatic atrial fibrillation in patients older than 70 years with complete atrioventricular (AV) block, normal left ventricular systolic function, and implanted dual chamber (DDD) pacemaker.

**Methods:**

Hundred and eighty six patients with complete AV block were admitted over one year to the Sisters of Mercy University Hospital. The study recruited patients older than 70 years, with no history of atrial fibrillation, heart failure, or reduced left ventricular systolic function. All the patients were implanted with the same pacemaker. Out of 103 patients who were eligible for the study, 81 (78%) were evaluated. Follow-up time ranged from 12 to 33 months (average±standard deviation 23 ± 5 months). Primary end-point was asymptomatic atrial fibrillation occurrence recorded by the pacemaker. Atrial fibrillation occurrence was defined as atrial high rate episodes (AHRE) lasting >5 minutes. Binary logistic regression was used to identify the predictors of development of asymptomatic atrial fibrillation.

**Results:**

The 81 patients were stratified into two groups depending on the presence of AHRE lasting >5 minutes (group 1 had AHRE>5 minutes and group 2 AHRE<5 minutes). AHRE lasting >5 minutes were detected in 49 (60%) patients after 3 months and in 53 (65%) patients after 18 moths. After 3 months, only hypertension (odds ratio [OR], 17.63; *P* = 0.020) was identified as a predictor of asymptomatic atrial fibrillation. After 18 months, hypertension (OR, 14.0; *P* = 0.036), P wave duration >100 ms in 12 lead ECG (OR, 16.5; *P* = 0.001), and intracardial atrial electrogram signal amplitude >4 mV (OR, 4.27; *P* = 0.045) were identified as predictors of atrial fibrillation.

**Conclusion:**

In our study population, hypertension was the most robust and constant predictor of asymptomatic atrial fibrillation after 3 months, while P wave duration >100 ms in 12-lead ECG and intracardial atrial signal amplitude were predictors after 18 months.

Atrial fibrillation is the most common cardiac arrhythmia, which is associated with high morbidity and mortality, primarily due to cerebrovascular thromboembolic accidents and heart failure (1). Atrial fibrillation can be either symptomatic or asymptomatic. The incidence and prevalence of asymptomatic atrial fibrillation in the general population and in patients who have a dual chamber permanent pacemaker (PPM) is unknown. Previous studies have shown that 90% of the patients with an implanted dual chamber PPM and previously documented atrial fibrillation are asymptomatic since they lack irregular ventricular rate (2).

It is well known that age is an important risk factor for the development of atrial fibrillation (3,4). It is also known that the majority of elderly people with atrial fibrillation die of stroke or heart failure (5).

Given the assumption that asymptomatic atrial fibrillation can contribute to those events, the question is whether we can stratify patients according to their risk of asymptomatic atrial fibrillation by using simple accessible methods. The first estimates of the asymptomatic atrial fibrillation incidence were published in 1994 and reported a ratio of asymptomatic to symptomatic atrial fibrillation of about 12:1 (3,6). Studies investigating patients with stroke or transient ischemic attack found atrial fibrillation in about 15%-28% of patients, depending on the monitoring method used, and excluding those with previously known atrial fibrillation (7,8).

The Atrial Fibrillation Follow-up Investigation of Rhythm Management study, which compared the rate vs rhythm control in participants with atrial fibrillation older than 65 years, showed a trend toward higher mortality in the rhythm control arm. Stroke as significant contributor to mortality in this group was explained by warfarin withdrawal and asymptomatic atrial fibrillation occurrence (9).

Comparative advantage of patients with implanted pacemaker is that they are under rhythm monitoring permanently, thus leading to detection of asymptomatic atrial fibrillation (10). In this study, we proposed that analyzing 12-lead ECG, natriuretic peptide values, and pacemaker recordings can help to identify patients who are at higher risk of developing asymptomatic atrial fibrillation and complications of this arrhythmia. The aim of this study is to evaluate the predictors of asymptomatic atrial fibrillation in patients with complete atrioventricular (AV) block and implanted PPM.

## Patients and methods

### Patients

A total of 186 consecutive patients with complete AV block were admitted to the Department of Cardiology at the Sisters of Mercy University Hospital in Zagreb, Croatia between September 1, 2005 and September 1, 2006. Overall, 103 patients were older than 70 years (77 years, range 71-94 years) and thus eligible for the participation in the study. Table 1 shows the demographic data of the patients. Of the total 103 patients, 13 refused to participate and 9 were lost to follow-up.

**Table 1:** Key characteristics of 81 patients with atrial fibrillation included in the study on incidence and predictors in patients older than 70 y with complete atrioventricular block and dual chamber pacemaker implantation*^†^

	No. (%) of patients with asymptomatic atrial fibrillation at:				
	baseline	3 mo		18 mo	
Patient characteristic		yes (n = 49)	no (n = 32)	yes (n = 53)	no (n = 28)
Male sex	53 (65)	34 (69)	19 (59)	37 (70)	16 (57)
Body mass index (mean ± standard deviation)	25.6 ± 1.9	25.7 ± 1.8	25.6 ± 2	25.6 ± 1.9	25.7 ± 1.2
Comorbidities:					
hypertension	71 (88)	45 (92)	26 (81)	49 (93)	22 (79)
diabetes mellitus	16 (20)	10 (20)	6 (19)	4 (8)	6 (21)
ECG, mean ± standard deviation:					
QRS width (ms)	105 ± 32	105 ± 3	106 ± 31	108 ± 3	102 ± 33
P wave width (ms)	99 ± 25	104 ± 3†	92 ± 19	106 ± 3†	85 ± 15
Echocardiographic parameters:					
LVEF>50%	81 (100)	49 (100)	32 (100)	53 (100)	28 (100)
diastolic dysfunction	22 (27)	14 (29)	8 (25)	16 (30)	6 (21)
interventricular septum (mm in diastole), mean ± standard deviation	12.7 ± 4.7	13.1 ± 5.5	12.2 ± 2.9	13 ± 5.4	12.2 ± 3
left atrium (mm, PLAX in systole), mean ± standard deviation	42.3 ± 3.7	42 ± 4.3	42.7 ± 2.8	42.2 ± 4	42.4 ± 3.3
left ventricle (mm, PLAX in diastole), mean ± standard deviation	51.1 ± 4.3	51.8 ± 4.7	49.9 ± 3.2	51.6 ± 4.9	50.1 ± 2.6
B type natriuretic peptide (mean ± standard deviation)		63.5 ± 73.9	56.5 ± 63.1	66.9 ± 83	53.2 ± 84.3
atrial natriuretic peptide, (mean ± standard deviation)		34.8 ± 18.6	38.9 ± 17.7	37.1 ± 22.4	35.2 ± 17.4
Therapy:					
beta blockers		12 (25)	10 (31)	14 (26)	8 (29)
ACE inhibitors		35 (71)	23 (71.9)	39 (73.6)	19 (68)
ventricular pacing >70% of time		38 (78)	21 (66)	40 (76)	19 (68)

*Abbreviations: LVEF – left ventricular ejection fraction; PLAX – parasternal long axis.

†*P* < 0.05 vs no asymptomatic atrial fibrillation (independent samples *t* test).

Inclusion criteria for this prospective study were complete AV block, age older than 70 years, and dual chamber PPM. Exclusion criteria were history of atrial fibrillation, previous heart surgery, systolic and diastolic heart failure, hyperthyroidism, reduced left ventricular systolic function estimated using left ventricular ejection fraction <50%, measured by Simpson (11) and Teichholz method (12), mitral stenosis of any degree, left atrial dilatation (greater than 50 mm measured from the parasternal long axis), moderate or severe mitral regurgitation, and renal failure. A signed informed consent for permanent pacemaker implantation and for the participation in the study was obtained. The study was approved by Ethics Committees of the hospital and the Zagreb University Medical School.

## Methods

The study included patients with complete AV block who received PPM. Indications for permanent pacemaker implantation were made according to the ACC/AHA/NASPE 2002 Guideline Update for Implantation of Cardiac Pacemakers and Antiarrhythmia Devices (13). All patients had a dual chamber PPM (SIGMA 303 DDDR, Medtronic, Minneapolis, MN, USA) implanted using cephalic or subclavian approach on the non-dominant hand side. Atrial lead was implanted in the right atrial appendage and ventricular lead in the right ventricular apex using active fixation leads. In all patients, pacemaker was programmed in DDDR mode with the same lower rate of 60 bpm, without any arrhythmic interventional algorithm available.

Several variables were considered as potential predictors of atrial fibrillation. Some of them are already known risk factors, such as age, diabetes, hypertension, and left atrium diameter (4). In addition, we wanted to test the association of the following factors with the atrial fibrillation: atrial electrogram characteristics, brain natriuretic peptides concentrations, and cumulative pacing rate. Parameters evaluated were P wavelength, sex, age, arterial hypertension, diabetes mellitus, angiotensin-converting enzyme (ACE) inhibitors, beta blockers, left atrium diameter in systole (measured in parasternal long axis – PLAX), diastolic dysfunction (grade II or higher), intracardiac atrial signal amplitude >4 mV and width >50 ms, cumulative ventricular pacing >70%, cumulative dual chamber pacing >20%, B-type natriuretic peptide (BNP), atrial natriuretic peptide (ANP), body mass index, and QRS width. We defined asymptomatic atrial fibrillation as atrial high rate episodes (AHRE) detected by the pacemaker, lasting for more than 5 minutes, as described in previous studies (14). In all study patients, the atrial bipolar sensitivity was programmed to 0.5 mV, and the AHRE diagnostic was programmed ON (with cut-off rate at 180bpm).

### Clinical evaluation and blood sampling

During the hospital stay, a complete medical history and physical examination were obtained for every patient. Standard 12-lead ECG, chest x-ray before and after PPM implantation, echocardiography, and standard laboratory tests were also performed. Blood (complete blood count, blood urea nitrogen, creatinine, electrolytes, glucose, creatine kinase, lactate dehydrogenase, troponin T levels, prothrombin time and activated partial thromboplastin time, C reactive protein, acid-base status, lipid profile) and urine were sampled for routine analysis and the tests were performed by the central hospital laboratory. Blood pressure was measured each day during the hospital stay and then at each follow-up visit. Blood pressure was measured according to the Practice Guidelines of the European Society of Hypertension for clinic, ambulatory, and self blood pressure measurement and the 2003 European Society of Hypertension – European Society of Cardiology guidelines for the management of arterial hypertension (15,16). Blood pressure was measured using mercury sphyngomanometer on both arms with the patient sitting, with back support, legs uncrossed, and the arm supported at heart level. Hypertension was defined as blood pressure higher than 140/90 mm Hg measured two times at least 24 hours apart (16). All patients were instructed about possible atrial fibrillation symptoms before discharge from the hospital and were asked to keep a log of their symptoms.

### Follow-up

Patients were followed-up in the outpatient clinic every three months. On each of the visits, standard physical status was obtained with blood pressure measurement as well as 12-lead ECG recording (ECG was recorded at standard 25 mm/s speed as well as 50 mm/s for more accurate measurements of P and R wave duration) and pacemaker interrogation (Medtronic CareLink programmer, model nr. 2090). Electrocardiographic parameters (P and R wave) were evaluated by a single observer on standard 12-channel ECG with 50 mm/s speed of recording (a median value of 3 consecutive beats was taken), and intracardiac measurements were performed during the pacemaker examination (atrial intracardiac electrogram duration and amplitude in unipolar and bipolar mode, taking a median value of 3 consecutive beats). Atrial sensing was adjusted as 30% to measured P wave amplitude with the intention to avoid atrial over-sensing and under-sensing. Patients were both interviewed and given a short questionnaire regarding symptoms. Blood was collected for atrial and brain natriuretic peptides. The upper limit of normal for BNP was <18.4 pg/mL and for ANP was <43 pg/mL according to manufacturer's recommendations. Both concentrations were determined by an immunoradiometric assay (SHIONORIA ANP and SHIONORIA BNP in vitro test; CIS Bio International, Gif-Sur-Yvette, Cedex, France).

### Statistical analysis

Distribution of values of individual variables was determined using Kolmogorov-Smirnov test and appropriate parametric and nonparametric tests were applied for the further analysis. Chi-square test was used to test the differences in qualitative variables between the groups. Independent samples *t* test was used as univariate method to assess the differences in the quantitative variables (age, body mass index, QRS, and P wave width, IVS, left atrium, left ventricle, BNP, ANP) between symptomatic and asymptomatic groups. To estimate the influence of clinical and other factors on the development of asymptomatic atrial fibrillation, binary logistic regression was conducted. *P* < 0.05 was considered to be significant. Software Statistica V8.0 (StatSoft, Inc., Tulsa, OK, USA) was used for the analysis.

## Results

The 81 patients who were eligible for this study underwent pacemaker implantation and were subsequently monitored over 12 to 33 months (average and standard deviation: 23 ± 5 months) and stratified into two groups depending on the duration of AHRE. The average age of the patients was 81 ± 6 years, 53 were male (65%), 71 (88%) had a history of hypertension, and 16 (20%) had a history of diabetes (baseline data summarized in Table 1). All patients had left ventricular ejection fraction ≥50%. After 3 months, 49 (60%) patients had AHRE, while the remaining 32 (40%) had no AHRE (Table 1). After 18 months, AHRE lasting for more than 5 minutes were detected in additional 4 patients so that 53 (65%) patients developed asymptomatic atrial fibrillation (Table 1). Univariate analysis showed that the only significant difference between the two groups was detected for native P wave duration >100 ms (*P* = 0.023 after 3 months and *P* < 0.001 after 18 months).

The multivariate analysis performed by binary logistic regression (Table 2) showed that the only significant predictor of developing atrial fibrillation after 3 months was arterial hypertension (odds ratio [OR], 17.63; 95% confidence interval [CI], 1.57-197.54) (Cox and Snell R^2^ = 31%, Nagelkerke R^2^ = 43%). The analysis of the 18-month follow-up identified 3 predictors of atrial fibrillation: arterial hypertension (OR, 14.0;95% CI, 1.19-163.45), P wave width greater than 100 ms in the standard 12-lead ECG (OR, 16.5; 95% CI, 2.97-91.69), and amplitude of intracardiac atrial potential >4 mV (OR, 4.27; 95% CI, 1.03-17.70) (Cox and Snell R^2^ = 20%, Nagelkerke R^2^ = 27%).

**Table 2:** Predictors of asymptomatic atrial fibrillation occurrence in patients (n = 81) included in the study on incidence and predictors in patients older than 70 y with complete atrioventricular block and dual chamber pacemaker implantation at 3 and 18 mo follow-up

	Follow up, odds ratios (95% confidence intervals)	
Monitored parameter	3 mo	18 mo
P wave width ≥100 ms	1.90 (0.55-6.50)	16.51(2.97-1.69)^†^
Age	1.00 (0.93-1.07)	1.03 (0.96-1.11)
Male sex	2.11 (0.69-6.49)	2.05 (0.57-7.45)
Arterial hypertension	17.63 (1.57-197.84)*	14.00 (1.19-165.43)^†^
Diabetes mellitus	1.15 (0.24-5.54)	2.04 (0.40-10.29)
ACE inhibitors	0.22 (0.03-1.46)	0.22 (0.03-1.69)
Beta blockers	0.75 (0.21-2.65)	1.03 (0.23-4.58)
LA diameter in systole (PLAX)	0.90 (0.77-1.07)	0.91 (0.76-1.08)
Diastolic dysfunction (grade II or more)	0.50 (0.12-2.08)	0.90 (0.17-4.65)
Intracardiac atrial signal amplitude >4 mV	0.63 (0.18-2.22)	4.27 (1.03-17.70)
Intracardiac atrial signal width >50 ms	1.00 (0.20-5.08)	3.17 (0.45-22.08)
Cumulative ventricular pacing >70%	0.40 (0.08-1.95)	0.72 (0.009-5.72)
Cumulative dual chamber pacing >20%	0.26 (0.04-1.79)	0.40 (0.08-1.95)
B type natriuretic peptide	1.01 (1.00-1.02)	1.00 (0.99-1.01)
Atrial natriuretic peptide	0.96 (0.93-1.00)*	0.99 (0.95-1.02)
Body mass index	1.04 (0.79-1.37)	0.90 (0.65-1.25)
QRS width	1.00 (0.98-1.02)	0.99 (0.97-1.02)

*Abbreviations: LA – left atrium; PLAX – parasternal long axis.

†*P* < 0.05 vs no atrial fibrillation (binary logistic regression).

## Discussion

We found that the incidence of asymptomatic atrial fibrillation (defined as AHRE lasting more than 5 minutes) was 65%. The multivariate analysis showed that the strongest predictor of asymptomatic atrial fibrillation in the patients with complete AV block and implanted permanent pacemaker was arterial hypertension. Low R^2^ values in logistic regression are the norm and this presents a problem when reporting their values to an audience accustomed to seeing linear regression values. Although some authors do not recommend routine publishing of R^2^ values from fitted logistic regression models, there are different opinions because higher percentage of explained variance is strongly connected to the number of predictors used in the model (17). It is much more important to know what we measure rather than to have the range of R^2^ values similar to those of linear regression (18). Other predictors of atrial fibrillation after 18 months were P wave width measured on standard ECG and intracardiac atrial signal amplitude.

Previous studies have shown an even greater incidence of atrial arrhythmias in patients with implanted pacemakers. The A-HIRATE study showed that the incidence of AHRE was 89% in patients with previous atrial tachyarrhythmias and 49% in patients with no history of atrial tachyarrhythmias (19). Quirino et al (20) showed that the incidence of atrial fibrillation was 74%. Both of these studies showed that most of the episodes were asymptomatic. Quirino et al also showed low sensitivity and positive predictive value of symptoms in detecting atrial fibrillation with dual chamber pacemakers, although this study investigated different group of patients (sick sinus syndrome).

According to previous reports, and European Society of Cardiology Guidelines for the management of atrial fibrillation, implantable devices can detect atrial fibrillation appropriately, particularly when the cut-off point for duration of AHRE>5 minutes are used (8,21). Also, as it was reported in the A-HIRATE study, pacemaker diagnostic algorithms showed high sensitivity and specificity, as well as positive predictive value for atrial tachyarrhythmias (19).

Our study shows a relatively high incidence of asymptomatic atrial fibrillation. Since all our patients were over 70 years old, 88% of them had arterial hypertension, and 20% had diabetes, the risk of stroke for those with proven atrial fibrillation is high.

Bearing in mind that the predictive value of pacing diagnostics for atrial fibrillation is high and that previous studies showed low sensitivity and positive predictive value of the symptoms to detect atrial fibrillation (20), our study indicates that when AHRE are found in a high risk population oral anticoagulation should be considered as a treatment option.

In contrast to some studies that suggested ACE inhibitors as effective non-antiarrhytmic drugs in preventing atrial fibrillation, our study failed to prove their influence on atrial fibrillation (22). In our study, hypertension was identified as the strongest predictor of atrial fibrillation. Although hypertension is not the strongest predictor of atrial fibrillation in general population, it is the most common underlying disease in patients with atrial fibrillation (23,24). Also, we measured cumulative ventricular (>70%) and cumulative dual chamber pacing (>20%). Both of these parameters did not increase the risk of asymptomatic atrial fibrillation although one would expect it. Possible explanation for these findings is the small number of patients and a relatively short follow-up. Moreover, we included very homogenous group of patients with no signs of heart failure, which could have introduced bias into our results.

Another predictor of atrial fibrillation in our study was P wave width measured on standard ECG. Different electrocardiographic markers have been proposed for the assessment of risk for atrial fibrillation: maximum P wave duration, P index, P wave dispersion, and morphological changes of the P waves (25-28). Although none of these was clearly conclusive in our study population, P wave width appeared as potential predictor of atrial fibrillation after 18 months. We find this result particularly important since this parameter is far more available than signal average P wave duration proposed by other authors (29). In addition, P wave duration is generally accepted as reliable non-invasive marker of atrial conduction and its prolongation has been associated with the history of atrial fibrillation. Furthermore, P wave duration was previously reported as a predictor of atrial fibrillation occurrence after cardiac surgery and predictor of transition from paroxysmal to permanent atrial fibrillation (25,26).

We also found that P wave ampitude >4 mV on intracardiac recording could serve as a predictor of asymptomatic atrial fibrillation in patients after 18 months. This could partially be explained by the fact that patients with better atrial lead sensing and higher P wave amplitudes could have better detection of atrial arrhythmias.

Some other characteristics that could be expected to have an effect on atrial fibrillation occurrence, such as cumulative ventricular or cumulative dual chamber pacing rate, BNP and ANP levels, were not associated with asymptomatic atrial fibrillation occurrence in our study. This is probably due to a relatively small number of patients. As previously discussed, this study analyzed very homogenous group of patients and thus the results cannot be applied to patients with other indications for permanent cardiac pacing. Other conditions, such as heart failure or severe mitral regurgitation, can be stronger predictors of atrial fibrillation but those patients were not included in our study.

In conclusion, we showed that hypertension was the most robust and constant predictor of asymptomatic atrial fibrillation occurrence, while P wave duration >100 ms in 12-lead ECG and intracardial atrial signal amplitude could serve as predictors after 18 months. High incidence of asymptomatic atrial fibrillation mandates close and careful follow-up of patients with PPM, and in patients with high risk the utilization of oral anticoagulation should be considered. Future prospective studies on larger number of patients are needed to confirm and support our findings.
